# The discrepancy between admission and discharge diagnoses: Underlying factors and potential clinical outcomes in a low socioeconomic country

**DOI:** 10.1371/journal.pone.0253316

**Published:** 2021-06-15

**Authors:** Samar Fatima, Sara Shamim, Amna Subhan Butt, Safia Awan, Simra Riffat, Muhammad Tariq

**Affiliations:** 1 Department of Medicine, Section of Internal Medicine, Aga Khan University Hospital, Karachi, Pakistan; 2 Section of Gastroenterology, Department of Medicine, Aga Khan University Hospital, Karachi, Pakistan; 3 Department of Medicine, Aga Khan University Hospital, Karachi, Pakistan; Juntendo University Urayasu Hospital, JAPAN

## Abstract

**Objective:**

The discrepancy between admission and discharge diagnosis can lead to possible adverse patient outcomes. There are gaps in integrated studies, and less is understood about its characteristics and effects. Therefore, this study was conducted to determine the frequency, characteristics, and outcomes of diagnostic discrepancies at admission and discharge.

**Design and data sources:**

This retrospective study reviewed the admitting and discharge diagnoses of adult patients admitted at Aga Khan University Hospital (AKUH), Internal Medicine Department between October 2018 and February 2019. The frequency and outcomes of discrepancies in patient diagnoses were noted among Emergency Department (ED) physician versus admitting physician, admitting physician versus discharge physician, and ED physician versus discharge physician for the full match, partial match, and mismatch diagnoses. The studied outcomes included interdepartmental transfer, Intensive Care Unit (ICU) transfer, in-hospital mortality, readmission within 30 days, and the length of stay. For simplicity, we only analyzed the factors for the discrepancy among ED physicians and discharge physicians.

**Results:**

Out of 537 admissions, there were 25.3–27.2% admissions with full match diagnoses while 18.6–19.4% and 45.3–47.9% had mismatch and partial match diagnoses respectively. The discrepancy resulted in an increased number of interdepartmental transfers (5–5.8%), ICU transfers (5.6–8.7%), in-hospital mortality (8–11%), and readmissions within 30 days in ED (14.4%-16.7%). A statistically significant difference was observed for the ward’s length of stay with the most prolonged stay in partially matched diagnoses (6.3 ± 5.4 days). Among all the factors that were evaluated for the diagnostic discrepancy, older age, multi-morbidities, level of trainee clerking the patient, review by ED faculty, incomplete history, and delay in investigations at ED were associated with significant discrepant diagnoses.

**Conclusions:**

Diagnostic discrepancies are a relevant and significant healthcare problem. Fixed patient or physician characteristics do not readily predict diagnostic discrepancies. To reduce the diagnostic discrepancy, emphasis should be given to good history taking and thorough physical examination. Patients with older age and multi-morbidity should receive significant consideration.

## Introduction

Maintaining a high accuracy rate between admitting and discharge diagnosis is challenging and has significant clinical, financial, and legal implications in case of discrepancy. One of the significant consequences of discrepant diagnoses during hospitalization is an increased length of stay [1]. Diagnosis is the process of determining which illness is responsible for a patient’s symptoms and signs. The history, physical examination, and investigations play a fundamental role in making an effective initial diagnosis.

Often one or more diagnostic tests are also done during the process. It allows the physician to make medical decisions about treatment and prognosis. The diagnosis made at the time of admission is the foundation of an initial course of treatment provided by the physicians and accounts for the difference in the care provided during the hospital stay. The discrepancy in diagnosis that develops during the hospital course can lead to patient dissatisfaction, complaints, and litigations [2].

Gaps in the diagnosis at the time of admission are due to many reasons, including incomplete investigations and clerical errors [3]. When a patient is admitted through the Emergency Department (ED), ED physicians usually make the initial diagnosis based on the patient’s presentation at arrival, physical examination, and limited laboratory workup. This initial diagnosis may also differ from the diagnosis made by admitting physicians in the ward. Hence, the diagnosis can change during hospital stay, especially in complex cases leading to a variation in diagnosis at the time of discharge [4].

In a study conducted by GH Lim et al., 13.3% of patients admitted through ED to the inpatient services had discrepant (unmatched) diagnoses [5]. In another study, up to 71.4% of the diagnoses were fully or partially matched between ED and admitting services with 66.8% diagnostic accuracy for admission in Medicine Specialty. The accuracy was 76.9% and 90.3% for surgical and orthopedic patients [3]. A retrospective study conducted at a tertiary care hospital in Pakistan revealed that the total number of mismatched diagnoses in the department of Medicine through ED was 41% (1995), 37% (2000), and 14% (2007) [6].

Discrepant diagnoses may have several undesirable consequences such as increased length of hospital stay, in-hospital mortality [7, 8], and increased financial burden [9]. In a low socioeconomic country like Pakistan where a typical bread earner’s income is limited, the discrepancy in diagnoses may contribute to additional psychological and financial challenges for patients and their families. The data regarding the discrepancy between admission and discharge diagnoses from a low socioeconomic country like Pakistan are meager. The factors associated with it may also differ due to differences in the distribution of various diseases, healthcare setups, and facilities. The healthcare setups here consist of both public and private institutions with variable limited financial coverage from government and insurance agencies.

Moreover, in our institution, the length of stay and readmission rate is used as a quality indicator for faculty appraisal and both could be affected by mismatched or missed diagnoses. Therefore, it is imperative to study the frequency and factors associated with discrepant diagnoses. This will help to improve the quality of care provided to the patients and implement the necessary steps to enhance our education and training programs hence improving the professional competencies of medical practitioners.

Therefore, the current study was conducted to determine the frequency of diagnostic discrepancies at admission (diagnoses made by ED and admitting physician) and discharge from the hospital. We also aimed to determine the characteristics of patients and diagnoses leading to a higher rate of diagnostic discrepancies, along with the outcomes (interdepartmental transfer, ICU transfer, in-hospital mortality, readmission rate, and length of stay) associated with it.

## Material and methods

### Study design/Data source

This retrospective study was conducted at the Aga Khan University Hospital (AKUH). Established in 1985, AKUH is a 740 bedded, one of the largest university hospitals, and it provides a broad range of secondary and tertiary care. It caters to a variety of cases referred from all over Pakistan and offers both undergraduate and postgraduate training programs in multiple subspecialties.

Admissions to the Internal Medicine specialty in AKUH are either elective or via the ED. AKUH ED caters to patients of all ages. In Pakistan, emergency medicine as a specialty was first recognized and approved for residency training in 2010 by the College of Physicians and Surgeons of Pakistan (CPSP), a public regulatory college that oversees postgraduate medical education and professional development. It was the first time in Pakistan that dedicated emergency doctors provided emergency care to all the patients coming to the ED. Prior to this emergency care was managed by residents from all specialties (rather than ED residents) and a few casualty medical officers. AKUH has a structured emergency medicine training program and ED doctors are well-trained and experienced in delivering emergency care.

Patients visiting AKUH emergency are first triaged and then admitted to the relevant area of ED. There are separate areas for adult and pediatric patients in ED. ED physicians assigned in the adult ED area, evaluate all patients, then admit the patients in the relevant specialties, and whenever needed, relevant subspecialties (like surgery or Gynecology teams) are also involved. However, all Internal Medicine patients are admitted directly by ED physicians in ward under the care of Internal Medicine physicians. In this manuscript, we have only included those adult patients admitted in Internal Medicine specialty from ED or electively.

The workup done in ED depends on the patient’s initial assessment. In general, some baseline workup including complete blood count, creatinine, electrolytes, and random blood sugar is immediately sent after the admission of the patient to ED. Chest X-ray and ECG are also done for those patients who present with cardiorespiratory symptoms. Likewise, abdominal imaging is done instantly depending upon the indication. For example, if the patient is suspected of the diagnosis of mesenteric ischemia or peritonitis (in which the management is completely different), the CT scan and ultrasound for diagnosis is done without any delay (within 4 hours).

### Eligibility criteria and data collection

Patients above 18 years of age admitted electively from clinics or through the emergency department (ED) under the care of Internal Medicine specialty from October 2018 to February 2019 were identified using the International Classification of Diseases (ICD) coding system. A total of 1835 cases were identified. Systematic random sampling was done by selecting every 3rd patient for review. The population studied represented a sample size of 537 hospitalizations. The direct admissions to ICU, other subspecialties, and transfers from other hospitals were excluded from the study. Hospital-acquired infections and iatrogenic complications during hospital stay were excluded as the above conditions affect the discharge diagnosis.

The diagnoses were defined as specific if they pinpointed a particular pathological process involving one (or more than one) particular organ. Discrepancies were assessed by comparing the degree of mismatch between admitting and discharge diagnoses. Our main outcome, i.e. the diagnostic discrepancies were divided into three groups labeled as Full match, Partial match, and Mismatch. ‘Full match’ was considered when the initial and discharge diagnoses were the same. ’Partial match’ meant the initial diagnosis correlated to a certain extent with the final diagnosis, while ‘Mismatch’ was considered when the final discharge diagnosis did not correlate to the provisional diagnosis.

#### Example of partially matched diagnosis

An ED diagnosis of diabetic foot partially matched with a final diagnosis of critical limb ischemia. Another example is an initial diagnosis of Metabolic acidosis partially matched with the final diagnosis of Diabetic Ketoacidosis.

#### Example of mismatched diagnosis

An ED diagnosis of acute appendicitis mismatched with a final diagnosis of viral fever and acalculous cholecystitis. Another example is an initial diagnosis of Meningitis mismatched with the final diagnosis of viral fever.

The frequency and outcomes of discrepant diagnoses were noted among ED physician versus admitting physician, admitting physician versus discharge physician, and ED physician versus discharge physician for the full match, partial match, and mismatch diagnoses. The secondary outcomes studied included interdepartmental transfer, ICU transfer, in-hospital mortality, readmission within 30 days, and the length of stay.

In our hospital, both the assessment and diagnosis of the patient by the resident as well as the attending physician are documented in the patient’s file. In this study, we used the ICD codes only to pick up the Internal Medicine admissions. The admitting and discharge diagnoses mentioned in our study are not through ICD codes, but they are the diagnoses made by the physician and written down in the file (on admission and on discharge).

We have compared the discrepancy in the discharge diagnoses not only with the diagnoses made by ED physicians but also have compared the diagnoses among the admitting Internal Medicine physicians and discharging Internal Medicine physician.

For simplicity, we only analyzed the factors (such as age, gender, comorbid conditions, physicians level in ED and ward, incomplete history, missed physical examination, admission diagnoses based on symptoms, change in patient’s condition after admission) for the diagnostic discrepancy among ED physician and discharge physician in terms of the full match, partial match, and mismatch. The study was approved by the Ethical Review Committee (ERC) at the AKUH.

### Patient and public involvement statement

This was a retrospective study conducted by reviewing medical charts. It did not involve any live interview or interaction with the patients directly. The Ethical exemption was taken from the ERC of AKUH, Karachi, Pakistan before the commencement of this study. The results of this study will be disseminated by publishing the manuscript in a scientific journal.

### Statistical analysis

The data were entered and analyzed using the Statistical Package for Social Science SPSS (Release 16.0, standard version, copyright © SPSS; 1989–02). A descriptive analysis was done and the results were presented as mean ± standard deviation or Median (IQR) for continuous variables and number (percentage) for categorical variables. Analytical analysis was done according to the study objectives. For comparative univariate analysis, we used ANOVA for continuous variables and Pearson chi-square test for categorical variables. All p-values were two-sided and considered as statistically significant if < 0.05.

## Result

### Baseline characteristics of study subjects

A total of 537 admissions were included in the study. The mean age was 58.4 ± 18.9 years (range 18–93 years). The majority were females 52.3% and the most common comorbid condition of the study population was hypertension (53.1%), diabetes mellitus (43.9%) followed by ischemic heart disease (19.4%), and chronic kidney disease (13.2%) **(Table 1)**.

**Table 1:** Characteristics of the study population (n = 537).

	Mean ± SD or n (%)
**Age (years)**	58.4 ± 18.9
**Gender**	
**Male**	256(47.7)
**Female**	281(52.3)
**Comorbid conditions**	
**Yes**	362(67.4)
**Diabetes Mellitus**	
**Yes**	236(43.9)
**Hypertension**	
**Yes**	285(53.1)
**IHD**	
**Yes**	104(19.4)
**Asthma**	
**Yes**	20(3.7)
**COPD**	
**Yes**	22(4.1)
**CLD**	
**Yes**	15(2.8)
**CKD**	
**Yes**	71(13.2)
**History of TB**	
**Yes**	21(3.9)
**Malignancy**	
**Yes**	26(4.8)
**Cerebrovascular Accident**	
**Yes**	25(4.7)

**DM**: Diabetes Mellitus**, HTN:** Hypertension, **IHD**: ischemic heart disease, **COPD**: chronic obstructive airway disease, **CLD:** chronic liver disease **CKD:** Chronic kidney disease, **TB:** Tuberculosis

### Frequency and outcome associated with discrepant diagnoses

Comparing the diagnostic discrepancy between ED and Admitting physician group, out of 537 patients 45.3% (n = 243) had a partial match while full match and mismatch were found in 27.2% (n = 146) and 19.4% (n = 104) respectively. Out of 537, 287 cases had different admitting and discharge physicians, therefore the diagnostic discrepancy between ED and discharge physician was also reviewed and revealed 47.9% (n = 257), 25.3% (n = 136), and 18.6% (n = 100) partial match, full match, and mismatched diagnoses respectively. Not applicable were those cases that were admitted to the Internal Medicine specialty electively. Considering the different admitting and discharge physicians, the diagnostic discrepancies between them were also reviewed. In this case, the frequency for discrepant diagnoses was found to be 32.6% (n = 175) for partial match and 3.4% (n = 18) for mismatch **(Table 2)**.

**Table 2:** Frequency of diagnostic discrepancy.

	n = 537 (%)
**The diagnostic discrepancy between ED physician and Admitting physician**	

**Mismatch**	104(19.4)
**Full Match**	146(27.2)
**Partially Match**	243(45.3)
**Not Applicable**	44(8.2)
**The diagnostic discrepancy between Admitting physician and Discharge physician**	

**Mismatch**	18(3.4)
**Full Match**	344(64.1)
**Partial Match**	175(32.6)
**The diagnostic discrepancy between ED physician and Discharge physician**	

**Mismatch**	100(18.6)
**Full Match**	136(25.3)
**Partial Match**	257(47.9)
**Not Applicable**	44(8.2)

In the case of mismatch (common to all three categories), there was an increased number of interdepartmental transfers (5–5.8%), ICU transfers (5.6–8.7%), in-hospital mortalities (8–11%), and readmissions within 30 days (14.4%-16.7%). While in case of a partial match, the outcome had more implications with increased frequency of interdepartmental transfers (8–8.6%), ICU transfers (7.4–8.2%), in-hospital mortalities (9.5–13.7%), and readmissions within 30 days (14%-15.2%). A statistically significant difference was observed for the length of stay in the ward with the longest stay in the case of partial matched diagnoses (6.3 ± 5.4 days) **(Table 3)**. Significantly higher mortality was also observed when the diagnosis was partially matched between admitting and discharge physicians (p-value 0.009).

**Table 3:** Outcomes associated with diagnostic discrepancy.

The diagnostic discrepancy between ED and Admitting physician				
	Mismatch (n = 104)	Full Match (n = 146)	Partial Match (n = 243)	*p*-value
**Interdepartmental Transfer**				
**Yes**	6(5.8)	5(3.4)	21(8.6)	0.12
**No**	98(94.2)	141(96.6)	222(91.4)	
**ICU transfer**				
**Yes**	9(8.7)	5(3.4)	18(7.4)	0.18
**No**	95(91.3)	141(96.6)	225(92.6)	
**In-hospital mortality**				
**Yes**	9(8.7)	11(7.5)	23(9.5)	0.80
**No**	95(91.3)	135(92.5)	220(90.5)	
**Readmitted within 30 days**				
**Yes**	15(14.4)	16(11.0)	37(15.2)	0.48
**No**	89(85.6)	130(89.0)	206(84.8)	
**Length of stay in ED (hours)**	9.8 ± 5.8	10.2 ± 5.8	9.8 ± 5.4	0.73
**Length of stay in ward (days)**	5.9 ± 5	4.2 ± 3.6	6.1 ± 5.2	<0.001
	**The Diagnostic discrepancy between Admitting and Discharge physician**			
	**Mismatch (n = 18)**	**Full Match (n = 344)**	**Partial Match (n = 175)**	***p*-value**
**Interdepartmental Transfer**				
**Yes**	1(5.6)	22(6.4)	14(8.0)	0.77
**No**	17(94.4)	322(93.6)	161(92.0)	
**ICU transfer**				
**Yes**	1(5.6)	19(5.5)	14(8.0)	0.54
**No**	17(94.4)	325(94.5)	161(92.0)	
**In-hospital mortality**				
**Yes**	2(11.1)	20(5.8)	24(13.7)	0.009
**No**	16(88.9)	324(94.2)	151(86.3)	
**Readmitted within 30 days**				
**Yes**	3(16.7)	45(13.1)	26(14.9)	0.80
**No**	15(83.3)	299(86.9)	149(85.1)	
**Length of stay in ED (hours)**	11.2 ± 5.4	10 ± 5.7	9.5 ± 5.5	0.34
**Length of stay in ward (days)**	5.3 ± 4.9	5.0 ± 4.9	6.5 ± 5.0	0.007
	**The Diagnostic discrepancy between ED and Discharge physician**			
	**Mismatch (n = 100)**	**Full Match (n = 136)**	**Partial Match (n = 257)**	***p*-value**
**Interdepartmental Transfer**				
**Yes**	5(5.0)	6(4.4)	21(8.2)	0.28
**No**	95(95.0)	130(95.6)	236(91.8)	
**ICU transfer**				
**Yes**	7(7.0)	4(2.9)	21(8.2)	0.13
**No**	93(93.0)	132(97.1)	236(91.8)	
**In-hospital mortality**				
**Yes**	8(8.0)	8(5.9)	27(10.5)	0.29
**No**	92(92.0)	128(94.1)	230(89.5)	
**Readmitted within 30 days**				
**Yes**	16(16.0)	16(11.8)	36(14.0)	0.64
**No**	84(84.0)	120(88.2)	221(86.0)	
**Length of stay in ED (hours)**	9.8 ± 5.7	10.3 ± 6.0	9.8 ± 5.4	0.67
**Length of stay in ward (days)**	5.3 ± 4.3	4.1 ± 3.4	6.3 ± 5.4	<0.001

### Factors associated with the diagnostic discrepancy between ED and discharge physician

The average age of our patients was 59.5 ± 19.1 years. A statistically significant diagnostic discrepancy was found among elderly patients with the oldest group of patients in the partially matched group. The majority of the patients were above 50 years, so there were more comorbid conditions in all three groups. In patients with more than two comorbid conditions, the proportion of partially matched diagnoses were highest with a frequency of 62.9% [p value of <0.001] **(Table 4)**.

**Table 4:** Factors leading to diagnostic discrepancy.

	The diagnostic discrepancy between ED and Discharge physician; n = 493				
	Total (n = 493)	Mismatch (n = 100)	Full Match (n = 136)	Partial Match (n = 257)	*p*-value
**Age, in years**	59 ± 19.1	55.5 ± 19.3	53.1 ± 20.7	63.4 ± 17	<0.001
**Gender**					
**Male**	228(46.2)	40(17.5)	69(30.3)	119(52.2)	0.26
**Female**	265(53.8)	60(22.6)	67(25.3)	138(52.1)	
**Comorbid**					
**No**	108(21.9)	28(25.9)	47(43.5)	33(30.6)	<0.001
**1–2**	164(33.3)	34(20.7)	45(27.4)	85(51.8)	
**>2**	221(44.8)	38(17.2)	44(19.9)	139(62.9)	
**Physician level in ED (Clerking)**					
**Intern**	41(8.3)	14(34.1)	4(9.8)	23(56.1)	0.03
**Resident**	452(91.6)	86(19)	132(29.2)	234(51.7)	
**Reviewed by ED Consultant**					
**No**	173(35.0)	42(24.3)	30(17.3)	101(58.4)	0.001
**Yes**	320(64.9)	58(18.1)	106(33.1)	156(48.7)	
**Consultant Level of ED Physician**					
**Senior Instructor**	190(38.5)	34(17.9)	65(34.2)	91(47.8)	0.02
**Assistant Professor**	65(13.2)	13(20)	20(30.8)	32(49.2)	
**Associate Professor**	65(13.2)	11(16.9)	21(32.3)	33(50.8)	
**Not reviewed**	173(35.1)	42(24.3)	30(17.3)	101(58.4)	
**Consultant Level of Admitting Physician**					
**Senior Instructor**	255(51.7)	54(21.2)	69(27.1)	132(51.8)	0.24
**Assistant Professor**	143(29)	26(18.2)	46(32.2)	71(49.7)	
**Associate Professor**	51(10.3)	15(29.4)	10(19.6)	26(51.0)	
**Professor**	44(8.9)	5(11.4)	11(25.0)	28(63.6)	
**Discharge diagnoses made by the same Physician as admitting**					
**No**	264(53.5)	46(17.4)	75(28.4)	143(54.2)	
**Yes**	229(46.5)	54(23.6)	61(26.6)	114(49.8)	0.23
**Consultant Level of Discharging Physician**					
**Senior Instructor**	235(47.4)	47(20)	62(26.4)	126(53.6)	
**Assistant Professor**	150(30.4)	29(19.3)	48(32)	73(48.7)	0.08
**Associate Professor**	55(11.2)	18(32.7)	9(16.4)	28(50.9)	
**Professor**	53(10.8)	6(11.3)	17(32.1)	30(56.6)	
**Complete History in ED**					
**No**	169(34.3)	45(26.6)	27(16)	97(57.4)	<0.001
**Yes**	324(65.7)	55(17)	109(33.6)	160(49.4)	
**Complete Physical Examination in ED**					
**No**	117(23.7)	27(23.1)	30(25.6)	60(51.3)	0.66
**Yes**	376(76.3)	73(19.4)	106(28.2)	197(52.4)	
**Delay in diagnostic workup in ED**					
**No**	483(98)	94(19.5)	134(27.2)	255(52.8)	0.006
**Yes**	10(2.0)	6(60)	2(20)	2(20)	
**Admission Diagnoses based on initial symptoms in ED**					
**No**	18(3.7)	5(27.8)	7(38.9)	6(33.3)	0.26
**Yes**	475(96.3)	95(20)	129(27.2)	251(52.8)	
**Change in Patients Condition after Admission in Ward**					
**No**	77(15.7)	13(16.9)	19(24.7)	45(58.4)	0.48
**Yes**	415(84.3)	87(21)	116(28)	212(51.1)	

Most of the patients in the ED were clerked by ED residents. However, the diagnostic discrepancy was higher when the patients were clerked by interns as compared to residents (p-value 0.03) with the highest discrepancy in the partial (56.1%) and mismatch (34.1%) group. Out of 493 patients admitted, 173 (35%) were not reviewed by ED faculty leading to the highest diagnostic discrepancy of 58.4% in the partially matched group (p-value 0.001). However, the proportion of fully matched diagnosis was higher (33.1%) when the patients were reviewed by ED faculty.

On further stratification, it was noted that most (38.5%) of the patients were seen by an ED Senior instructor. When comparisons were done based upon the level of an attending physician who had reviewed the patient in the ED, the partial match and mismatch in the case of senior instructor was 47.8% and 17.9% respectively, while it was 49.2%, 20% for Assistant Professor and 50.8%, 16.9% for Associate Professor respectively [p value of 0.02]. The diagnostic mismatch was lowest when the patient was reviewed by an Associate Professor in the ED. The proportion of full match and partial match was higher and significant when the complete history was taken in ED (<0.001) while physical examination had no impact on reducing the discrepant diagnoses. The absence of delay in the diagnostic workup in ED resulted in higher proportions of the partial match (52.8%) and full match (27.2%) diagnoses (p-value 0.006).

The discrepancy among admitting and discharging physicians in the ward was also reviewed. Most of them had full matched diagnoses if admitting and discharging physicians remained the same. However, the difference was not statistically significant.

## Discussion

Comparing the diagnoses at admission and discharge is not only a good measure of diagnostic accuracy but also a quality measure. Maintaining this diagnostic accuracy is a huge challenge [4]. A significant increase in hospital stay has been observed in case of a discrepancy in diagnosis at admission and discharge [1]. Literature for discrepant diagnoses in terms of frequency and cause analysis for low middle-income countries are limited. Hence, in this study, we have reported the frequency and the factors leading to a discrepant diagnosis. Besides, we have also assessed the relationship between discrepant diagnoses and the level of residents and treating physicians from a country where well-established systems to maintain such data were lacking.

Previous studies have analyzed frequency for the diagnostic discrepancy using ICD-9-M coding (International Classification of Diseases, Ninth Revision, and Clinical Modification). In the majority of such studies, ICD coded diagnoses entered at the time of admission and discharge were used for analysis and estimating the discrepant diagnoses. However, ICD coding is subject to error as there are limited coded diagnoses, and most of the time comorbid conditions or symptom-based diagnoses are available on the system. Also, the comparison of accuracy will be altered if more than two diagnoses are present [1, 6, 8, 10, 11]. Taking into consideration the methodology of previous studies and its limitation, in this study a thorough review of the patient’s medical charts, as well as electronic data were carried out. The diagnoses documented by the ED and admitting physicians on the file and the assessments documented by residents were also recorded.

The frequency of diagnostic discrepancy found in this study was comparable to other studies. Approximately 17% unmatched and 83% partial/full matched diagnoses have been reported in a study conducted by Chiu H et al. [3]. In another study, 13.3% unmatched diagnoses and 86.7% matched diagnoses have been observed [4, 5]. Although the frequency of mismatch cases was closely comparable with these studies, full and partially matched diagnoses were reported together in these studies. However, in our study both partial and full matched diagnoses were reported separately to reflect a real work-based situation analysis. The level of diagnostic discrepancy observed by Tricia et al, Cristina et al. and James et al. are even much higher than those shown in other researches which could be because they included patients from all specialties [1, 8, 11].

In literature, it has been noted that in-hospital length of stay increases with discrepant diagnosis (4.2 days as compared to 3.4 days without discrepant diagnoses) [1]. The length of stay found in this research is higher as compared to other studies. This could be due to the older age and multimorbidity of patients admitted to Internal Medicine specialty along with the lack of rehabilitation services in our part of the world. In terms of the interdepartmental transfer, GH Lim et al. found that unmatched diagnoses (18.8%) resulted in more interdepartmental transfer as compared with matched diagnoses (5.1%) [5]. This is comparable to the present study, as there was an increase in the number of interdepartmental transfers resulting from discrepant diagnoses. However, we also looked into some other outcomes associated with discrepant diagnoses that have not been evaluated before like ICU transfer, in-hospital mortality, and readmission rates. The other difference in the outcome of this study is that partially matched diagnoses had more implications than the mismatched diagnoses.

Some studies have assessed the diagnostic discrepancy between different specialties. It was observed that the diagnostic quality for patients with general medicine was marginally lower than that of general surgery (82.9% for General Surgery and 77.6% for General Medicine) [10]. To reduce discrepant cases in general medicine patients, other studies, including this study have concluded that good history and early investigations are among the key factors [3, 11]. Although there was no correlation between physical examination and discrepant diagnoses in this study. Other studies have contradictory observations.

In summary, there is a definitive need to reduce the diagnostic discrepancy. It is a known consideration that both the period spent by the patient and the tests performed during the hospital course may lead to a different diagnosis at the time of discharge. However excellent clinical assessment techniques including history taking and physical examination remain the most essential and rewarding diagnostic tools in diagnostic accuracy. Much attention should be paid to patients who are aged and suffering from multi-morbidity. To reduce the discrepancy and to arrive at an accurate diagnosis, the treating physician should mention a few particular differential diagnoses during their first assessment [12]. This ability to construct more than one possible differential diagnosis should also be emphasized to the undergraduate and postgraduate trainees during the treating physician rounds. It is a well-recognized factor that the patient’s actual diagnosis is the one diagnosis under which the patient improved enough to be discharged [7, 13]. Lack of proper documentation is one of the important factors that lead to the discrepant diagnosis. It is therefore important to improve the documentation practices [14, 15].

Our study does carry some limitations like other studies. It was a single-center study evaluating diagnostic discrepancy while focused on patients from a single specialty. There were errors in documentation in discharge summaries, however, all such gaps were reviewed by reviewing the patient’s medical records thoroughly. This study did not show any causation as this is a descriptive study. Similar studies in the future from other centers including other subspecialties will help to gauge the consistency of the results and to develop strategies to improve this important quality measure.

## Conclusion

Diagnostic discrepancies are a relevant and significant healthcare problem in patients admitted through the emergency room into the ward. Diagnostic discrepancies are not readily predictable by fixed patient or physician characteristics. Among all the factors that were evaluated for the diagnostic discrepancy, older age, multi-morbidities, level of trainee clerking the patient, review by ED faculty, incomplete history, and delay in investigations at ED were found to be associated with significant discrepant diagnoses. The longer lengths of hospital stay and increased mortality in certain cases were observed as the most serious outcomes associated with the discrepant diagnoses.

## Supporting information

S1 File (SAV)

## References

[1] Johnson T, McNutt R, Odwazny R, Patel D, Baker S. Discrepancy between admission and discharge diagnoses as a predictor of hospital length of stay. J Hosp Med. 2009;4(4):234–9. doi: 10.1002/jhm.453 19388065

[2] Leape LL, Brennan TA, Laird N, Lawthers AG, Localio AR, Barnes BA, et al. The nature of adverse events in hospitalized patients: results of the Harvard Medical Practice Study II. New England journal of medicine. 1991;324(6):377–84. doi: 10.1056/NEJM199102073240605 1824793

[3] Chiu H, Chan K, Chung C, Ma K, Au K. A comparison of emergency department admission diagnoses and discharge diagnoses: retrospective study. Hong Kong Journal of emergency medicine. 2003;10(2):70–5.

[4] McNutt R, Johnson T, Kane J, Ackerman M, Odwazny R, Bardhan J. Cost and quality implications of discrepancies between admitting and discharge diagnoses. Quality Management in Healthcare. 2012;21(4):220–7. doi: 10.1097/QMH.0b013e31826d1ed2 23011068

[5] Lim G, Seow E, Koh G, Tan D, Wong H. Study on the discrepancies between the admitting diagnoses from the emergency department and the discharge diagnoses. Hong Kong Journal of Emergency Medicine. 2002;9(2):78–82.

[6] Shahid M, Hameed K, Iqbal R, Afzal O, Nakeer R, Razzak J. Accuracy of diagnosis and relationship with quality of emergency medicine training program. Journal of the College of Physicians and Surgeons Pakistan. 2012;22(5):342. doi: 05.2012/JCPSP.342343 22538048

[7] Hautz WE, Kämmer JE, Hautz SC, Sauter TC, Zwaan L, Exadaktylos AK, et al. Diagnostic error increases mortality and length of hospital stay in patients presenting through the emergency room. Scandinavian journal of trauma, resuscitation and emergency medicine. 2019;27(1):54. doi: 10.1186/s13049-019-0629-z 31068188 PMC6505221

[8] Eames J, Eisenman A, Schuster RJ. Disagreement between emergency department admission diagnosis and hospital discharge diagnosis: mortality and morbidity. Diagnosis. 2016;3(1):23–30. doi: 10.1515/dx-2015-0028 29540045

[9] Mahieu L, Buitenweg N, Beutels P, De Dooy J. Additional hospital stay and charges due to hospital-acquired infections in a neonatal intensive care unit. Journal of hospital Infection. 2001;47(3):223–9. 10.1053/jhin.2000.0852 11247683

[10] Goh S, Low B. Accident and Emergency Department Diagnosis-How Accurate are We? Singapore medical journal. 1996;37:24–30. 8783908

[11] Leske MC, Sorensen AA, Zimmer JG. Discrepancies between admission and discharge diagnoses in a university hospital. Medical care. 1978:740–8. doi: 10.1097/00005650-197809000-00004 682708

[12] Raz E, Cohen S, Benbassat J. Evaluation of diagnostic accuracy in the clinical setting. Israel journal of medical sciences. 1987;23(12):1177–80. 3440739

[13] Hautz SC, Schuler L, Kämmer JE, Schauber SK, Ricklin ME, Sauter TC, et al. Factors predicting a change in diagnosis in patients hospitalised through the emergency room: a prospective observational study. BMJ open. 2016;6(5):e011585. doi: 10.1136/bmjopen-2016-011585 27169743 PMC4874162

[14] Jencks SF, Huff ED, Cuerdon T. Change in the quality of care delivered to Medicare beneficiaries, 1998–1999 to 2000–2001. Jama. 2003;289(3):305–12. doi: 10.1001/jama.289.3.305 12525231

[15] Graff LG, Wang Y, Borkowski B, Tuozzo K, Foody JM, Krumholz HM, et al. Delay in the diagnosis of acute myocardial infarction: effect on quality of care and its assessment. Academic emergency medicine. 2006;13(9):931–8. doi: 10.1197/j.aem.2006.04.016 16894002

